# Hypophosphatasia: Clinical Clues and Management Considerations

**DOI:** 10.7759/cureus.80894

**Published:** 2025-03-20

**Authors:** Jason M Corless, Alan J Bartholomew, Joseph K Kluesner

**Affiliations:** 1 Internal Medicine, Wright-Patterson Medical Center, Wright-Patterson Air Force Base, USA; 2 Internal Medicine, Wright State University, Dayton, USA; 3 Rheumatology, Wright-Patterson Medical Center, Wright-Patterson Air Force Base, USA; 4 Endocrinology, Wright-Patterson Medical Center, Wright-Patterson Air Force Base, USA

**Keywords:** alkaline phosphatase (alp), bone mineralization, hypophosphatasia, hypophosphatemic osteomalacia, rare genetic diseases

## Abstract

This case report identifies typical manifestations of a patient with hypophosphatasia, a rare genetic condition in which mutations in tissue-non-specific alkaline phosphatase (TNSALP) enzymes cause low levels of alkaline phosphatase and defective bone mineralization. It explores common diagnostic clues from the history and laboratory evaluation, which can help clinicians identify the disorder. This case introduces a 49-year-old patient with a long history of fractures and dental abnormalities who was referred to endocrinology for evaluation of osteopenia. Further review of her laboratory data was noteworthy for a low level of alkaline phosphatase. Additionally, a low vitamin B6 level was measured, and genetic testing was ultimately diagnostic for hypophosphatasia. The patient was started on the anabolic agent teriparatide but was lost to subsequent follow-up. This case discusses additional management considerations, which are currently limited but continue to evolve, and cautions against bisphosphonate use in the setting of hypophosphatasia.

## Introduction

Hypophosphatasia is a rare genetic condition caused by various mutations of the *ALPL* gene. These mutations lead to defective tissue-non-specific alkaline phosphatase (TNSALP) enzymes. This enzyme is responsible for degrading inorganic pyrophosphate into inorganic phosphate, which is essential for hydroxyapatite, a compound needed for bone formation and mineralization [1]. Excess inorganic phosphate can also lead to pseudogout [2]. Additionally, TNSALP plays a role in vitamin B6 metabolism and is necessary for the entry of vitamin B6 into cells [1].

This case presents an example of adult hypophosphatasia and demonstrates important diagnostic clues, including the history of intraoperative fractures, teeth abnormalities, bone pain, low alkaline phosphatase levels, and elevated vitamin B6 levels. Given the rarity of the condition, research into the treatment of hypophosphatasia is limited but growing. Recombinant alkaline phosphatase is available and, although traditionally reserved for more severe pediatric forms of the disease, is becoming more widely used in adults as well as anabolic agents such as teriparatide [2,3]. Importantly, bisphosphonate use is contraindicated, given the risk of osteomalacia [2]. Clinicians should be aware of the diagnostic clues associated with the diagnosis of hypophosphatasia and the emerging considerations regarding its treatment.

## Case presentation

A 49-year-old postmenopausal woman presented to an endocrinology clinic for evaluation of osteopenia. Her medical and surgical history was notable for Roux-en-Y gastric bypass surgery, osteoarthritis with bilateral hip arthroplasty, and lumbar degenerative disc disease. She also had a notable history of severe osteoarthritis with chronic shoulder, hip, and back pain, as well as vague generalized achy pain. She had been evaluated by primary care, orthopedic surgery, neurosurgery, and pain management for these complaints. She also had a history of depression and ADHD, which was well-controlled at the time of initial evaluation.

A detailed history revealed that the patient had experienced multiple bone fractures. This included an intraoperative femur fracture during hip arthroplasty about eight years prior. She had been told by the surgeon that her bones were "very brittle," although no further description from the surgeon was available in the medical record. Her history was also notable for wrist fractures on multiple occasions after falls on an outstretched hand, as well as broken ribs after falling while cleaning a chandelier. She also had evidence of lumbar spinal compression fractures, which had been treated conservatively. She denied any fractures or dental issues as a child but did report that she had had multiple chipped, broken, and loose teeth since her gastric bypass surgery. These issues had culminated in the need for multiple dental implant surgeries as well as bone grafts, which had been unsuccessful, necessitating the use of dentures.

Immediately after her gastric bypass surgery, she reported she had been consistent with calcium and vitamin D supplementation but, over the last five years, had not been supplementing her diet. She reported lactose intolerance and had limited dairy intake.

She reported only limited remote tobacco use without current use. She denied any alcohol or other substance use. She reported some regular moderate aerobic exercise and strength training. Her family history was significant for a mother and sister with osteoporosis. Her mother had suffered a hip fracture. She had begun menopause within the last two years.

The patient's primary care provider obtained a bone mineral density (BMD) scan, given her history of gastric bypass surgery and history of multiple fractures and dental issues. The BMD scan was difficult to interpret given the presence of bilateral hip replacements as well as extensive degenerative sclerosis of the spine, both of which made the bone density at these locations uninterpretable. However, her bone density at the distal radius was 0.712 g/cm^2^ with a T-score of -1.9, indicative of osteopenia. The primary care provider recommended that the patient take 1200 mg of calcium carbonate and 800 IU of vitamin D3 daily. The patient was referred to endocrinology for further evaluation and consideration of medications to prevent osteoporosis and further complications.

Her physical exam was unremarkable, without skeletal abnormalities other than the use of dentures. She had a normal musculoskeletal range of motion and strength and a normal sclera. Her laboratory studies were most notable for a low alkaline phosphatase level with additional previously low measurements and mildly low vitamin D levels (Table 1). Low levels of alkaline phosphatase prompted the consideration of hypophosphatasia as a potential cause of her osteopenia, especially in the setting of frequent fractures. Vitamin B6 levels were measured and were found to be elevated. Her labs also showed normal parathyroid hormone, osteocalcin, and collagen cross-linked c-telopeptide levels, as well as normal 24-hour urine calcium (Table 1).

**Table 1:** Laboratory data at the time of initial evaluation

Test	Result	Reference Range
Sodium	140 mmol/L	136-145 mmol/L
Potassium	4.6 mmol/L	3.4-4.9 mmol/L
Chloride	103 mmol/L	98-107 mmol/L
Carbon dioxide	30 mmol/L	23-31 mmol/L
Blood urea nitrogen	14 mg/dL	7-25 mg/dL
Creatinine	0.71 mg/dL	0.6-1.3 mg/dL
Glucose	68 mg/dL	74-109 mg/dL
Alanine aminotransferase	28 U/L	7-52 U/L
Aspartate aminotransferase	21.7 U/L	13-39 U/L
Alkaline phosphatase	22 U/L (LOW)	34-104 U/L
Bone-specific alkaline phosphatase	5.2 mcg/L (LOW)	8.1-31.9 mcg/L
Bilirubin	0.5 mg/dL	0.3-1.0 mg/dL
Calcium	9.9 mg/dL	8.6-10.3 mg/dL
Vitamin D, 25-hydroxy	28.10 ng/mL (LOW)	30.0-100.0 ng/mL
Phosphate	4.7 mg/dL	2.5-5.0 mg/dL
Vitamin B6	179.7 mcg/L (HIGH)	2.0-32.8 mcg/L
Parathyroid hormone	72.2 pg/mL	12-88 pg/mL
Osteocalcin	20.2 ng/mL	9.4-47.4 ng/mL
Collagen cross-linked c-telopeptide	322 pg/mL	34-1037 pg/mL
24-hour urine calcium	236.8 mg/24 hours	100-300 mg/24 hours
Thyroid-stimulating hormone	1.12 mcIU/mL	0.45-5.330 mcIU/mL
Hemoglobin	14.2 g/dL	12.0-16.0 g/dL
White blood cell count	8.7 x 10^3^/mcL	4.6-9.4 x 10^3^/mcL
Platelets	334 x 10^3^/mcL	140-420 x 10^3^/mcL

Genetic testing was performed, showing a heterozygous mutation in the *ALPL* gene, with the pathogenic variant (p.Ala116Thr) consistent with hypophosphatasia [4-6]. The patient was started on calcium citrate 600 mg daily and vitamin D3 400 IU twice daily. Teriparatide 20 µg daily was initiated, and she was referred for genetic counseling.

**Figure 1:**
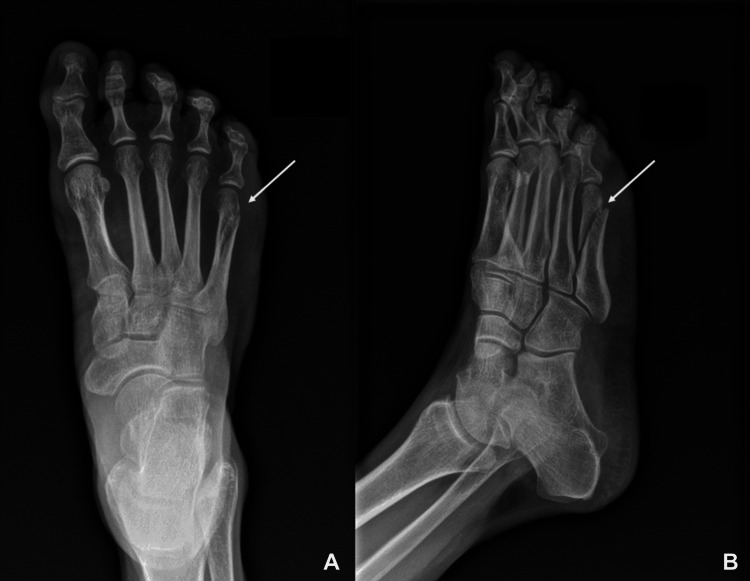
X-ray images of the fifth metatarsal fracture (A) Anteroposterior view; (B) oblique view

About 18 months after the patient was lost to the endocrinology clinic, the patient was seen within our health system in the orthopedics clinic for a fifth metatarsal fracture after an accidental fall. Images of the fracture are shown in Figure 1.

## Discussion

Hypophosphatasia is a rare genetic condition resulting from various mutations of the *ALPL* gene, which leads to defective TNSALP enzymes. This family of enzymes is responsible for the degradation of inorganic pyrophosphate into inorganic phosphate, which is needed for hydroxyapatite formation and bone mineralization. Ineffective bone mineralization presents clinically in adults as osteopenia with frequent fractures, loss of adult dentition, and bone pain. The absence of the enzyme also leads to the accumulation of the substrate inorganic pyrophosphate, an inhibitor of bone mineralization that can precipitate and build up in joints, causing pseudogout [1].

Additionally, the TNSALP enzyme plays a role in vitamin B6 metabolism. The active form of vitamin B6, pyridoxal-5-phosphate, is unable to diffuse across cell membranes without prior hydrolysis to the inactive form pyridoxal. The TNSALP enzyme performs this conversion, which permits pyridoxal to enter the cell, where it is enzymatically rephosphorylated back into the active form pyridoxal-5-phosphate [1]. Vitamin B6 plays a role in neurotransmitter synthesis, and seizures can be seen in some pediatric forms of the disease [7].

The *ALPL* gene has a high level of allelic heterogeneity, with more than 400 different mutations described [8]. This leads to a high variability of clinical presentation, ranging from mild to severe forms presenting from infancy to late adulthood. The wide spectrum of disease presentation has typically been divided into six classical forms: perinatal lethal, prenatal benign, infantile, childhood, adult, and odontohypophosphatasia [9].

The true prevalence of hypophosphatasia is difficult to determine given the rarity of the condition as well as its widely variable presentation, which often leads to misdiagnosis. A particularly high incidence has been found among Mennonite communities in Manitoba, Canada, with approximately one in 25 individuals carrying an *ALPL* gene mutation [10]. However, the general overall prevalence in North America is estimated to be much lower at 1:100,000 [8].

This case presents an example of adult hypophosphatasia and demonstrates important diagnostic clues regarding the clinical and biochemical manifestations of the disease. Adult hypophosphatasia is usually discovered around middle age and is mild compared to infantile and pediatric forms, which are typically more severe [10]. Common initial clinical manifestations include foot pain with recurrent metatarsal fractures and joint pain related to pseudogout. Hip pain can also occur with femoral fractures and pseudofractures [8,10]. Loss of dentition is common in many patients, and some will also recall a premature loss of deciduous teeth on further questioning [10]. Adult hypophosphatasia can become disabling due to recurrent musculoskeletal pain and recurrent fractures. Frustration among patients is often common due to a delay in diagnosis. Although more research is needed, some experts question whether derangements in the vitamin B6 pathway involved in neurotransmitter synthesis may also play a role in the high prevalence of depression, anxiety, and chronic pain in patients with hypophosphatasia [2]. Our patient had experienced multiple common manifestations of the disease, such as loss of adult dentition, multiple previous fractures, including an intraoperative femur fracture, metatarsal fractures, and chronic pain and anxiety.

The hallmark biochemical finding in hypophosphatasia is low alkaline phosphatase levels, which often correlate with disease severity [11]. Patients with hypophosphatasia will generally have consistently low levels on multiple occasions. When hypophosphatasia is suspected, other conditions such as hemochromatosis, malnutrition, hypothyroidism, vitamin D intoxication, multiple myeloma, and Wilson's disease should be considered as they can also rarely present with low alkaline phosphatase levels [8,12]. Other associations with low alkaline phosphatase levels include cardiac bypass surgery, bisphosphonate therapy, massive transfusion, and exposure to radioactivity [8].

Additional laboratory and radiologic clues can also be observed to diagnose hypophosphatasia. Elevated levels of vitamin B6 are common due to its inability to enter cells. Hyperphosphatemia and hypercalcemia can rarely occur due to impaired skeletal mineralization, although they are much more common in pediatric forms of the disease [9]. Osteopenia is often present on BMD measurement, although it is not necessary for the diagnosis, as many patients with hypophosphatasia have an increased risk for fracture despite an increase in bone density [8].

Ultimately, the diagnosis is confirmed using genetic analysis, looking for known mutations in the *ALPL* gene. These mutations can cause autosomal dominant or recessive inheritance patterns [1]. Most cases of adult hypophosphatasia are heterozygous mutations with over 400 known missense mutations. This wide variety of mutations leads to variable presentations of the disease, ranging from fetal death to fractures in adulthood, as seen in our patient [1,8]. Patients with mutations identified should be referred to genetic counseling.

Treatment of hypophosphatasia continues to evolve. In adults, the goals of therapy include reducing the risk of fracture as well as managing symptoms related to the disease, such as chronic joint and bone pain [2]. Low-impact exercise is often recommended as these patients are at an increased risk of fracture, and physical and occupational therapy can often be helpful [2,3]. Careful calcium and vitamin D supplementation when deficiencies are identified can help prevent secondary hyperparathyroidism and help mitigate fracture risk [2]. However, excess supplementation should be avoided because excess calcium supplementation is thought to contribute to calcium pyrophosphate precipitation [1]. Patients should have close orthopedic follow-up for the management of fractures with the understanding that many fractures fail to heal properly and may eventually require indwelling hardware for stabilization [3]. Pain is often managed well with non-steroidal anti-inflammatory drugs or local glucocorticoids [8]. Concomitant mental health concerns should also be addressed and managed appropriately, as these issues are common in this patient population.

Recombinant TNSALP, known as asfotase alfa, is commercially available and has been more widely studied in the pediatric population. It has been shown to rapidly improve skeletal, muscular, pulmonary, and cognitive development with rapid decreases in plasma levels of inorganic phosphate in children [8]. Its use in adults is less well-studied, although some investigations show promising early results with improved bone density, normalized TNSALP substrate levels, and improved function and quality of life [8]. It should be noted that the medication is still prohibitively expensive for most patients and often not covered by insurance [13]. The use of recombinant TNSALP in adults will hopefully increase as the medication becomes more widely studied in adults and affordability improves.

Some studies have evaluated the off-label use of anabolic agents such as teriparatide and found increases in bone density and decreases in fractures in some patients [2,13]. Teriparatide, a recombinant parathyroid hormone, is thought to stimulate osteoblasts and cause an increase in the synthesis of endogenous TNSALP [13]. The ability of teriparatide to elicit an increase in TNSALP synthesis is probably variable and dependent on the specific mutation, of which there are many possibilities. Our patient, in this case presentation, was started on teriparatide; however, she was eventually lost to follow-up, rendering it impossible to evaluate her response.

It should be noted that several treatment modalities have been found to be ineffective or even harmful. Early studies investigated the use of infusions of alkaline phosphatase-rich plasma in attempts to replace the deficient enzymes. However, these were unsuccessful, and recombinant forms of the TNSALP enzyme were eventually developed, as noted above [2]. Bone marrow and stem cell transplantation have also been evaluated, especially in pediatric forms of the disease, but with only limited success [9].

Finally, it should be noted that the diagnosis of adult hypophosphatasia is often missed or delayed as its presenting symptoms are similar to more common conditions such as age-related osteoporosis or other secondary causes of osteoporosis. With respect to treatment methods, it becomes especially important to distinguish between hypophosphatasia and the more common causes of osteoporosis. In contrast to more common causes of osteoporosis where bisphosphonates are first-line therapies, bisphosphonates are contraindicated in hypophosphatasia as they have been observed to cause worsening of underlying osteomalacia [9]. As noted above, pyrophosphate is a substrate of the TNSALP enzyme, and its accumulation leads to the inhibition of bone mineralization. This inhibition is enhanced by the presence of bisphosphonates, which are pyrophosphate analogs [8]. Thus, in all patients with decreased bone density, an alkaline phosphatase level should be measured prior to bisphosphonate therapy. Low alkaline phosphatase levels would warrant further evaluation for hypophosphatasia, which would prohibit the use of bisphosphonates.

## Conclusions

This case presented a patient with the typical clinical findings of adult hypophosphatasia, of which clinicians should be aware. It introduces a 49-year-old female patient with osteopenia, recurrent fractures, dental abnormalities, and bone pain. The diagnostic importance of the low alkaline phosphatase level present in the disease is highlighted as well as its value in early detection. The patient was also found to have elevated levels of vitamin B6, which is typical of the condition. Although hypophosphatasia is rare, recognition is important because treatment differs from that of typical patients with decreased bone density.

The patient, in this case, was treated with the anabolic agent teriparatide but was eventually lost to follow-up. Additional treatment considerations are discussed, including agents that are becoming more available, such as recombinant TNSALP. Ineffective or harmful therapies are also described. Notably, bisphosphonates should be avoided. Living with hypophosphatasia can be difficult and frustrating for patients, who often go undiagnosed. Increased awareness of the disorder and knowledge of the common findings, such as the low alkaline phosphatase levels, will allow for greater ability to care for patients with this genetic condition.
